# CrnnCrispr: An Interpretable Deep Learning Method for CRISPR/Cas9 sgRNA On-Target Activity Prediction

**DOI:** 10.3390/ijms25084429

**Published:** 2024-04-17

**Authors:** Wentao Zhu, Huanzeng Xie, Yaowen Chen, Guishan Zhang

**Affiliations:** College of Engineering, Shantou University, Shantou 515063, China; 21wtzhu@stu.edu.cn (W.Z.); 22hzxie@stu.edu.cn (H.X.); ywchen@stu.edu.cn (Y.C.)

**Keywords:** CRISPR/Cas9, deep learning, sgRNA, on-target, DeepSHAP

## Abstract

CRISPR/Cas9 is a powerful genome-editing tool in biology, but its wide applications are challenged by a lack of knowledge governing single-guide RNA (sgRNA) activity. Several deep-learning-based methods have been developed for the prediction of on-target activity. However, there is still room for improvement. Here, we proposed a hybrid neural network named CrnnCrispr, which integrates a convolutional neural network and a recurrent neural network for on-target activity prediction. We performed unbiased experiments with four mainstream methods on nine public datasets with varying sample sizes. Additionally, we incorporated a transfer learning strategy to boost the prediction power on small-scale datasets. Our results showed that CrnnCrispr outperformed existing methods in terms of accuracy and generalizability. Finally, we applied a visualization approach to investigate the generalizable nucleotide-position-dependent patterns of sgRNAs for on-target activity, which shows potential in terms of model interpretability and further helps in understanding the principles of sgRNA design.

## 1. Introduction

CRISPR/Cas9 (clustered regularly interspaced short palindromic repeats and CRISPR-associated system) is a promising genome-editing tool in biology. It consists of specifically edited single-guide RNA (sgRNA) and Cas9 protein in carrying nuclease activity [1]. The Cas9 protein can recognize protospacer adjacent motif (PAM) sequences under the guidance of sgRNA and introduce double-strand breaks of site-specific DNA into the target region, thus realizing site-specific region editing in the genome [2]. Binding and cleavage occur through the complementation of the 20 nt protospacer within the sgRNA with the target sequence in the genome, which is the upstream 3′ end of the PAM sequence. The success of CRISPR/Cas9 in prokaryotic host genetic engineering heavily relies on the activity of sgRNA. However, there are still significant limitations in the variability of sgRNA activity, leading to inconsistency in terms of on-target efficiency. Therefore, maximizing target site activity and accurately predicting sgRNA activity can enhance the safety of CRISPR-based experiments.

Numerous learning-based methods have been employed to predict sgRNA on-target activity. These methods are primarily divided into two categories: traditional machine-learning-based and deep-learning-based methods. The former methods require manual feature extraction before training the model. For instance, WU-CRISPR [3] utilizes structural features such as double-stranded structure stability, secondary structure stability, dinucleotide counts and trinucleotide counts for feature combination and uses support vector machine [4] for model training. However, the process of manual feature extraction requires domain experience, necessitating the design and adjustment of feature engineering for specific problems. Therefore, it is evident that traditional machine-learning-based methods have limitations when it comes to dealing with complex features and non-linear relationships.

Deep learning can automatically learn the multi-layered abstract representations of input features. It performs well in handling complex features and non-linear relationships. Convolutional neural networks (CNNs) [5] and recurrent neural networks (RNNs) are two commonly used architectures of deep learning [6]. CNNs perform well in the field of computer vision and have also gained widespread attention in sequence processing problems [7,8], which can identify longer-term patterns in sequences with less computational cost. For example, Xue et al. [9] proposed a CNN-based DeepCas9 method for on-target activity prediction, which skips the step of feature extraction from sequence objects by automatically learning sequence determinants and further identifying functional sgRNAs. On the other hand, RNNs, such as long short-term memory (LSTM) [10] and gated recurrent unit (GRU) [11], can update their internal states as they read input sequences, allowing them to capture interactions between units in the sequence. RNNs can extract high-dimensional feature representations in sgRNA sequences that are different from those of CNNs. For instance, Wang et al. [12] developed an RNN-based method called DeepHF for on-target activity prediction. It used LSTM to generate new patterns based on previously learned patterns and features of sgRNA. This characteristic allows it to capture the sequence information from sgRNA. Zhang et al. [13] proposed a hybrid architecture, C-RNNCrispr, which combines a CNN and an RNN for predicting sgRNA activity. It utilized the CNN to extract sequence features and applied bidirectional GRU (BiGRU) [14] to model the positional dependencies of sgRNA sequences in both forward and reverse orders. By integrating the multi-level representation of the CNN with a contextual understanding of BiGRU, C-RNNCrispr achieved good feature extraction capability, thus improving the model’s performance.

Recent studies have shown that incorporating a transfer learning strategy [15] by making use of existing data and experience between different tasks to improve the learning effect of new tasks can improve the performance of deep-learning-based methods. Fine-tuning [16] is a popular transfer learning strategy. For instance, Zhang et al. [17] used a large-scale benchmark dataset for pre-training and applied a fine-tuning strategy to boost the performance on small-scale datasets. Notably, most of the existing deep-learning-based on-target predictors were dataset-dependent. Thus, this calls for developing more accurate methods for predicting sgRNA activity.

Here, we proposed CrnnCrispr, an interpretable deep learning method that combines a CNN and an RNN for predicting sgRNA on-target activity. CrnnCrispr comprised two branches, namely a CNN branch and a BiGRU branch, which were responsible for abstract feature extraction from the encoded matrices using one-hot encoding and label encoding, respectively. The outputs of these two branches were combined and fed into the LSTM layers to learn the dependencies between the sequence features identified by the two branches. Subsequently, the outputs of the LSTM layers were flattened and input into three fully connected layers. The final output layer consisted of one neuron related to a regression score that highly correlates with sgRNA activity. Experiments demonstrated that our model achieved satisfactory accuracy and generalizability on nine public datasets with various scales (e.g., large-, medium- and small-scale datasets). Additionally, we performed a visualization approach to investigate how nucleotides at specific positions in the sgRNA affected the on-target activity learned by our CrnnCrispr model, which contributes to sgRNA design in practical use.

## 2. Results and Discussion

### 2.1. Ablation Study Shows the Importance of Convolution Module

We first conducted a comparative analysis of four models derived from our original CrnnCrispr model by modifying its components as follows (Table 1):

To analyze the impact of each module in our CrnnCrispr model for sgRNA on-target activity prediction, we conducted ablation experiments on the WT dataset. It was randomly divided into 85% as the training set and 15% as the testing set. We performed 5-fold cross-validation during the training phase. Compared with CrnnCrispr, the models applying only one-hot encoding (CrnnCrispr-onehot) or label encoding (CrnnCrispr-label) to encode the sgRNA sequences led to a slight decrease in prediction performance (Table 2). This indicates that both one-hot encoding and label encoding can help to extract the abstract features of sgRNA. Thus, using these two encoding methods for representing the inputs for two separate branches provides the model more informative sequence information, further allowing it to learn more complex and higher-level representations. Additionally, we observed that after removing the BiGRU branch, the CrnnCrispr-w/oBiGRU model achieved SCC and PCC values of 0.861 and 0.884, respectively. In addition, when only using the BiGRU branch to receive the encoded inputs, there was a significant decrease in SCC and PCC, with values of 0.836 and 0.865, respectively. This demonstrates the importance of the CNN module in our CrnnCrispr model. The CNN can effectively identify simple patterns in the sequence and generate complex patterns at higher levels. In addition, our standard CrnnCrispr achieved the best performance, with SCC and PCC values of 0.867 and 0.892, respectively. This implies that although 1D-CNN is not sensitive to the sequential order of the sequence and lacks global features, it compensates for this by incorporating high-dimensional features from BiGRU at higher levels. The subsequent architecture of the model captures both global and local representations, leading to improved performance. Together, these results indicate that the complete model structure achieved the optimal performance in all metrics.

### 2.2. Performance Comparisons of State-of-the-Art Methods on Datasets with Various Scales under 5-Fold Cross-Validation

**Figure 1:**
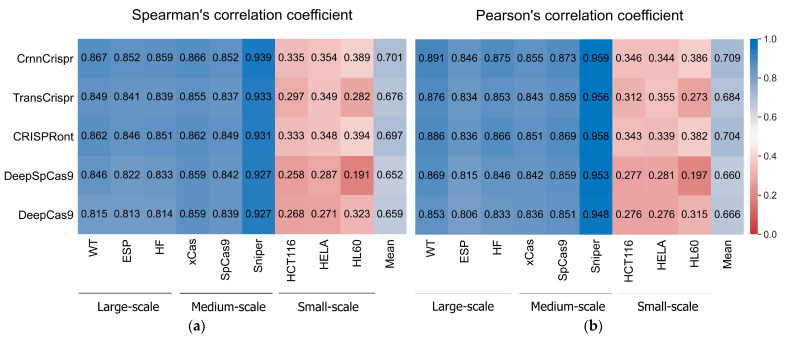
The heatmap shows (**a**) mean SCC and (**b**) mean PCC values of CrnnCrispr and four compared methods on nine datasets with three scales, including large-scale, medium-scale and small-scale datasets. The prediction methods are placed vertically, whereas the test datasets are arranged horizontally. Test datasets are classified by sample size.

To evaluate the performance of CrnnCrispr, we compared it with four existing methods (i.e., TransCrispr [18], CRISPRont [19], DeepSpCas9 [20] and DeepCas9 [9]) on nine public datasets. We note that TransCrispr consists of two model architectures, namely, BioNet and PureNet. The former incorporates both sequence features and biological features (e.g., melting temperature, free energy, GC content and secondary structure), and the latter is based solely on sequence features. For fair comparison, we only selected the PureNet architecture. Each dataset was randomly divided into a training set and a testing set according to the method described in Section 2.1. All the methods used the same parameter settings during the training phase. The averaged results of each metric of the 5-fold cross-validation across all the datasets are summarized in Figure 1.

We observed that CrnnCrispr achieved the highest SCC on all the datasets except for the HL60 dataset (Figure 1a). It achieved comparable performance with CRISPRont in terms of SCC. Overall, CrnnCrispr obtained the highest SCC of 0.701 (SD = 0.258) across all the datasets. Specifically, on the large- and medium-scale datasets, CrnnCrispr achieved average SCC values of 0.859 (SD = 0.008) and 0.886 (SD = 0.047), which were higher than the second-best CRISPRont by 0.006 and 0.005. The averaged SCC of CrnnCrispr on the three small-scale datasets was higher than that of CRISPRont by 0.01. Similarly, we also observed that CrnnCrispr achieved the highest PCC, with a mean value of 0.709 (SD = 0.264) on all nine datasets (Figure 1b). More specifically, it achieved averaged PCC values of 0.871 (SD = 0.023), 0.896 (SD = 0.056) and 0.359 (SD = 0.024) on the large-scale, medium-scale and small-scale datasets, respectively. Together, these observations suggest the effectiveness of CrnnCrispr for sgRNA on-target activity prediction on datasets with various scales. In addition, the methods trained and tested on the large- and medium-scale datasets gained better performance than those trained and tested with the small-scale datasets. It is expected that deep-learning-based predictors rely heavily on sufficient training datasets. There is still room for improving the prediction ability of these methods on small-scale datasets.

### 2.3. Transfer Learning Can Improve the Performance for Small-Scale Dataset Prediction

**Figure 2:**
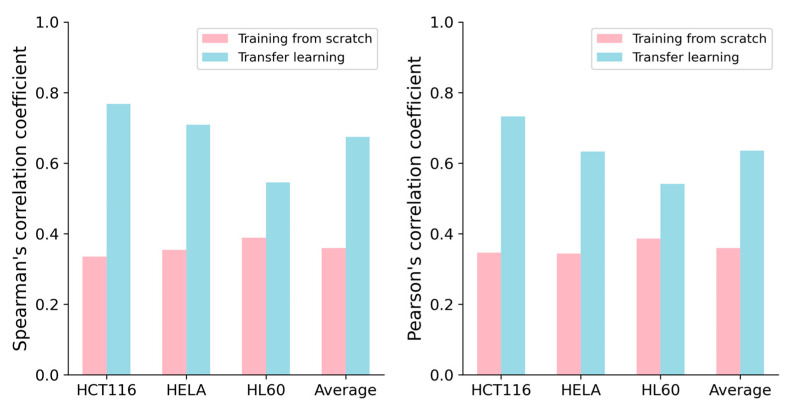
Performance comparison of CrnnCrispr training from scratch and transfer learning on three small-scale datasets (e.g., HCT116, HELA and HL60) under 5-fold cross-validation.

Previous studies have shown that transferring knowledge from experiments conducted on large-scale datasets to experiments on small-scale datasets through transfer learning [21] can lead to deep learning models with superior performance, such as C-RNNCrispr and CNN-XG [22]. Inspired by Zhang et al. [17], we applied transfer learning to improve the performance of our CrnnCrispr model on small-scale datasets. We used the benchmark dataset to pretrain our model. The benchmark dataset was randomly divided into a training dataset and testing dataset with a proportion of 8:2. Through 5-fold cross-validation, we obtained a well-trained pre-training model. We applied the fine-tuning strategy on the small-scale datasets. More specifically, we froze all the layers of the two branches in the model and utilized the pre-trained weights of the optimal base network. During the fine-tuning process, only the last two fully connected layers of the model were trained. We set the hyperparameters of batch size, learning rate and epoch with values of 0.00001, 500 and 500, respectively. Each small-scale dataset (HCT116, HELA and HL60) was divided into training data and testing data with a proportion of 9:1. The training data of these three datasets were combined to create a training set. We evaluated the performance of our model on the testing set of the interested cell line under 5-fold cross-validation. We observed that CrnnCrispr incorporating a fine-tuning strategy consistently enhanced the performance on all three small-scale datasets in terms of both SCC and PCC (Figure 2). It gained averaged SCC values of 0.768, 0.709 and 0.545 on the HCT116, HELA and HL60 datasets, which were 43.3%, 35.5% and 15.6% higher than the model training from scratch, respectively. Similarly, CrnnCrispr incorporating fine-tuning yielded a 27.6% improvement in the mean PCC on these three datasets. Together, these results showed that fine-tuning can significantly enhance the performance of CrnnCrispr on small-scale datasets.

We next explored whether the fine-tuning could enhance the performance of other methods. To this end, we pretrained the compared methods (e.g., TransCrispr, CRISPRont, DeepSpCas9 and DeepCas9) using the benchmark dataset and fine-tuned them on three small-scale datasets. Five-fold cross-validation tests were randomly performed, and the average values of the individual performances are summarized in Table 3. Overall, compared with the results of training from scratch (Figure 2), the methods incorporating fine-tuning could consistently improve the model performance. We observed that our CrnnCrispr performed best on these three small-scale datasets, reaching a mean SCC of 0.674 and PCC of 0.635. CRISPRont exhibited the highest PCC on the HCT116 and HELA datasets, with values of 0.735 and 0.649, respectively. TransCrispr achieved the highest PCC score (0.561) on the HL60 dataset. Together, these results illustrated that deep learning methods incorporating a fine-tuning strategy can significantly improve the prediction ability on small-scale datasets.

### 2.4. Generalizability Evaluation of Deep-Learning-Based Methods under a Leave-One-Cell-Out Procedure

In this section, we evaluated the generalizability of our CrnnCrispr for on-target activity prediction. For this purpose, we compared it with four existing methods, including TransCrispr, CRISPRont, DeepSpCas9 and DeepCas9, on the nine datasets with various cell types under a leave-one-cell-out cross-validation test. For each dataset, we divided it into a training set and a testing set following the procedure described in Section 2.2. For each dataset, we only used the remaining two datasets at the same scale as the training set (excluding the given interested training data) during model training. During the testing phase, we evaluated the performance using the given interested testing data. Taking the leave-WT-out procedure as an example, we trained the model by combining the training data from two other large-scale datasets, including ESP and HF (excluding the training data from WT), and evaluated the model on the testing data of the WT dataset.

**Figure 3:**
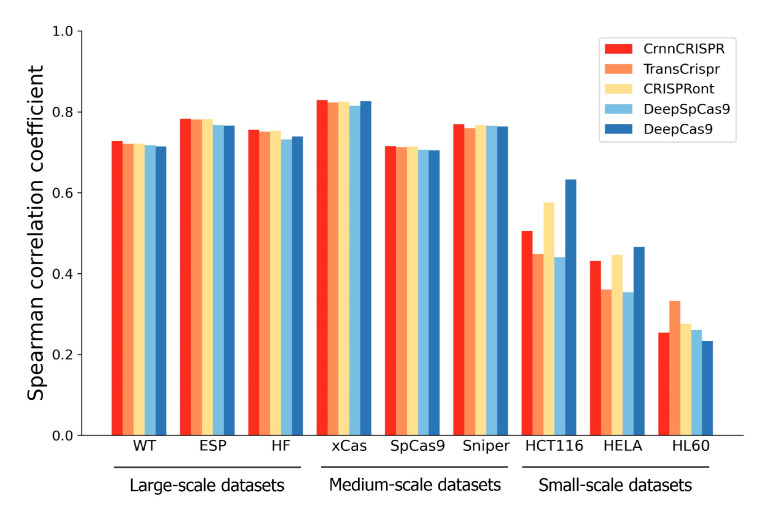
Performance comparison in terms of SCC of CrnnCrispr and four existing deep-learning-based methods on nine datasets with various scales under a leave-one-cell-out procedure.

We observed that CrnnCrispr, TransCrispr and CRISPRont achieved satisfactory performance on the large-scale datasets (Figure 3). On average, CrnnCrispr showed an SCC value of 0.756, which was 3% higher than the second-best CRISPRont. In addition, CrnnCrispr gained the best performance on the medium-scale datasets, with a mean SCC of 0.768, which was 2% higher than other compared methods. Moreover, it ranked third in terms of SCC for the small-scale datasets, which was slightly lower than CRISPRont and DeepCas9. A similar observation was also obtained in terms of PCC (Supplementary Figure S1), where the results show that CrnnCrispr achieved the best average performance on the large- and medium-scale datasets while ranking second on the small-scale datasets. Together, CrnnCrispr shows good generalizability with sufficient data, while there is room for improvement on small-scale scenarios.

### 2.5. Model Interpretability

Finally, we utilized a well-trained CrnnCrispr model to compute the contribution of nucleotides at each position to the activity of sgRNA and visualized the output, thus investigating the contribution of sequence patterns in sgRNA to its targeting activity. This was inspired by previous works (e.g., DeepHF and CNN-SVR [17]) that applied site importance analysis to explore the feature importance of all possible position-specific nucleotides. To compare the nucleotide contribution values across datasets of different data scales, we rescaled the output values using Z-score normalization. Nucleotides with Z-scores higher than 1 or lower than −1 contribute significantly to the activity of sgRNA.

**Figure 4:**
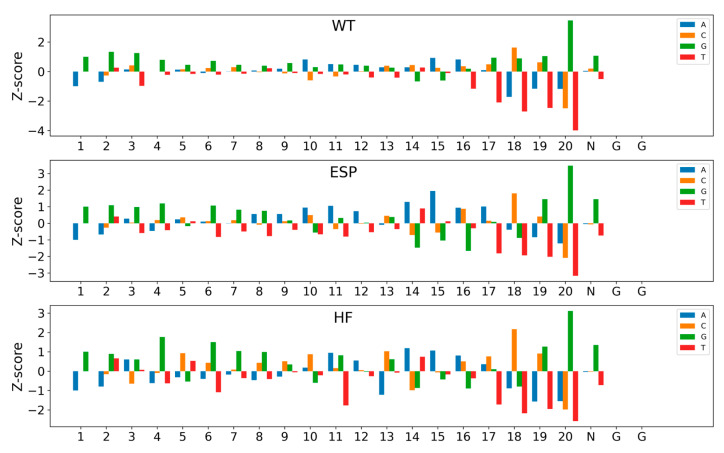
Impact of nucleotide composition of sgRNA activity on three large-scale datasets. Bars show the Z-scores of nucleotide frequency for each position. The numbers below represent the positions of the sequence.

Figure 4 depicts the visualization of the feature importance of position-specific nucleotides for three datasets (e.g., WT, ESP and HF) in the large-scale dataset. We observed that G had a significant positive contribution, while T had a noticeable negative contribution. This observation aligns with the findings from DeepHF, where Cas9 preferentially binds to sgRNAs containing purines but not pyrimidines. All three Cas9 nucleases (WT, ESP and HF) have a certain number of significant nucleotides, with 15, 20 and 20, respectively. Moreover, most of these nucleotides contribute to sgRNA activity. Nucleotide 20 was the most prominent site, where G had the strongest positive contribution and T had the strongest negative contribution. In addition, C_20 also showed a significant negative contribution. In contrast, the negative contribution of T_18 exceeded that of C_20 on the WT and HF datasets. In addition, several nucleotides, including G_1–4, G_6–8 and multiple A in the spacer region, positively impacted sgRNA activity, while T_16–21 resulted in low sgRNA expression.

Supplementary Figure S2 shows the influence of the nucleotide composition of sgRNA activity on three medium-scale datasets, including xCas, SpCas9 and Sniper. Similarly to the large-scale datasets, we also observed that G exhibited a significant positive contribution, while T exhibited a significant negative contribution. In contrast, G at positions 22 and 23 was the highest positive-contributing sites, while A, C and T at these two positions showed significant negative contributions. Supplementary Figure S3 depicts the visualizations of the feature importance of position-specific nucleotides for three small-scale datasets (HCT116, HELA and HL60). Similarly to the large-scale datasets, these three datasets with NGG PAM also showed that positions 22 and 23 had no impact on sgRNA on-target activity. Most of the significant nucleotides had the same contribution direction to sgRNA activity on the HCT116 and HELA datasets. The most prominent site was the nucleotide at position 18, where C exhibited the strongest positive contribution, while A showed the strongest negative contribution. Consistent with the conclusion obtained from Zhang et al. [17], C provided information at 3nt upstream of PAM, as cleavage sites are typically located around 3nt upstream of the PAM sequence [23]. On the HL60 dataset, significant nucleotides exhibited a different trend compared to the other two cell lines. G showed a positive contribution in most positions, while T displayed a negative contribution at most positions. The nucleotides at position 20 had the greatest impact on sgRNA activity. Specifically, G_20 demonstrated the strongest positive contribution, while T_20 exhibited the strongest negative contribution. Moreover, G at positions 14 to 16 showed a negative contribution, while T at these positions had a positive contribution. Similar results were also observed on the datasets HCT116 and HELA.

In addition, by analyzing the feature importance of position-specific nucleotides on nine datasets with three scales, we observed that positions 22 and 23 had no impact on the activity for the sequences with NGG PAM. On the contrary, in the case of Cas9 variant data with PAM sequences in any form, the influential sites affecting activity were predominantly concentrated at positions 22 and 23. Furthermore, the contributions of most significant nucleotides to sgRNA activity were consistent within each dataset scale. It is often observed that nucleotides adjacent to the PAM proximal end exert the most substantial impact on sgRNA activity.

### 2.6. Discussion

Accurately predicting CRISPR/Cas9 sgRNA on-target activity is crucial for understanding this system. Previous studies have shown that CNNs demonstrate outstanding performance in handling biological sequence problems [24]. Moreover, studies have explored the integration of regularization and pooling operations into CNNs to reduce the reliance on local features and enhance the generalization ability. Here, we proposed a model called CrnnCrispr, which combines a CNN and BiGRU for sgRNA on-target activity prediction. Specifically, we utilized a stacked CNN architecture without incorporating regularization or pooling operations. In addition, we employed a residual-like BiGRU structure where the embedding matrix was merged with the output of the first BiGRU layer and fed into the next layer, establishing a connection between low- and high-dimensional features. In addition, we employed the parallel architecture to extract contextual sequential features, which were subsequently concatenated at higher levels. Finally, dimensionality reduction was applied to the sequence features at different abstraction levels to retain the most effective features.

Our ablation study showed the effectiveness of the convolution module in our CrnnCrispr architecture for extracting the informative information of the sgRNA sequences. In addition, CrnnCrispr achieved the best performance compared to four state-of-the-art methods on nine public datasets with three scales. As there is shared information among the datasets, it can help deep learning models for training with insufficient data. By employing transfer learning and fine-tuning strategies, models can effectively utilize the shared information in the datasets. We found that applying fine-tuning could effectively improve the performance of our model for small-scale dataset prediction. We also found that the lighter networks such as CRISPRont and DeepCas9 consistently demonstrated superior generalizability on the small-scale datasets. This indicates that deeper networks or parallel architectures are more reliant on the amount of data, resulting in poor generalizability. In addition, we visualized the feature importance of all possible position-specific nucleotides learned by our CrnnCrispr for each dataset, which can help to clearly observe the characteristics of sgRNA activities of datasets with various scales, thus contributing to the design of sgRNA.

Furthermore, our results showed that nucleotide contribution in HL60 differed from the HCT116 and HELA datasets, which might be attributed to the fact that the latter two datasets are derived from tumor cells, while dataset HL60 is derived from a leukemia cell line. These cell lines have different genotypes and mutations that may affect the nucleotide contributions. In addition to the characteristics of the sgRNA sequence, the molecular environment of genomic editing may influence Cas9 activity. Deep learning models trained on hand-crafted cell-type-specific features (e.g., DNA methylation, H3K4me3 position information, chromatin accessibility and CTCF binding information) may contribute to the prediction accuracy. For example, Chuai et al. [25] proposed the DeepCRISPR model intended for on-target prediction using sgRNA sequence and epigenetic features such as DNA methylation information. The authors illustrated that adding cell-type-specific biological features can improve the performance. Similarly, Kim et al. [26] demonstrated that considering chromatin accessibility to create a deep-learning-based model can boost the prediction ability for on-target prediction. This study only considered sgRNA sequences. Consequently, there remains a need for more work to facilitate the analysis of on-target data by incorporating cell-type-specific features.

Our future work will focus on two areas. One area is about exploring methods for improving the generalizability of the model. Most existing deep-learning-based sgRNA on-target activity prediction methods demonstrate excellent performance on specific datasets. Therefore, this issue should be critically resolved when training on datasets with varying sample sizes and ranges of activities. The second area is about improving the model’s interpretability. Currently, there are many model architectures in the field of natural language processing that demonstrate outstanding performance in handling sequential problems. Incorporating biological sequence information into these architectures is worth exploring to enhance the model’s interpretability. Apart from the sequence of sgRNA, incorporating hand-crafted features into deep-learning-based architectures has been reported to give improved model interpretability, such as structure features, epigenetic features and thermodynamic features [12,25]. Future work should consider various informative sequence-derived features.

## 3. Materials and Methods

### 3.1. Data Resources

We used nine public datasets for model training and testing (Table 4), namely, WT [27], ESP [28], HF [29], xCas9 [30], SpCas9-NG [31], Sniper-Cas9 [32], HCT116 [33], HELA [33] and HL60 [34]. The first three datasets were processed by Wang et al. [12]. The authors performed a genome-scale screening and measured the sgRNA activity of two highly specific SpCas9 variants (eSpCas9(1.1) and SpCas9-HF1) and the wild-type SpCas9 (WT-SpCas9) in human cells. They obtained insertion–deletion rates for over 50,000 sgRNAs per nucleotide, covering around 20,000 genes. After removing the unedited sequences, we obtained insertion–deletion rates for three nucleotides, namely, WT, ESP and HF, with quantities of 56,887, 58,616 and 55,603, respectively. In addition, we selected the xCas9, SpCas9-NG and Sniper-Cas9 datasets from Kim et al. [35] as our experimental data. After removing duplicates, we obtained 37,738, 30,585 and 37,794 samples for each variant, respectively. Chuai et al. [25] integrated four independent validated human datasets of sgRNA cleavage efficiency. These experiment-based datasets were sourced from public datasets [33,34], encompassing sgRNA from 1071 genes across four different cell lines. We used the HCT116, HELA and HL60 datasets. By removing redundancies, the samples of these datasets were 4239, 8101 and 2076, respectively. According to the sample size, we divided them into three groups of large- (e.g., WT, ESP and HF), medium- (e.g., xCas9, SpCas9-NG and Sniper-Cas9) and small-scale (e.g., HCT116, HELA and HL60) datasets.

In addition, a previous study showed that the distal region of PAM had a high tolerance to sequence mismatch [23]. In addition, sgRNAs with two mismatches at the first two positions of the 5′ end hardly affected the cleavage efficiency [36]. Inspired by these studies, Chuai et al. [25] applied a data augmentation method and obtained a total of 180,512 sgRNAs with labels for model training, thus improving the performance. We used their augmentative dataset as a benchmark dataset for model pre-training when applying fine-tuning for small-scale dataset prediction.

### 3.2. Sequence Encoding

We use one-hot encoding and label encoding [37] for sequence encoding. Specifically, one-hot encoding was used to encode sgRNA sequences and as the input for the CNN branch. The input sequence was transformed into a matrix with a size of 4 × L, where 4 represents the number of nucleotide types (A, C, G and T), and L is the length of sgRNA. Each individual non-zero element in the matrix corresponded to the respective position of the nucleotide. Specifically, the nucleotides A, C, G and T in the sequence were encoded as [1, 0, 0, 0], [0, 1, 0, 0], [0, 0, 1, 0] and [0, 0, 0, 1], respectively. In addition, we incorporated label encoding to encode the sgRNA sequence and as input for the BiGRU branch. Specifically, sgRNA was converted into a numeric sequence. An integer, 1, was added at the beginning as the start bit padding. Each nucleotide (A, C, G and T) in the sequence was represented as an integer (2, 3, 4 and 5). The embedding weight matrix of dimension k was executed after the encoding, and each base was encoded into a vector of size k. The number sequence was mapped to a dense real-value space. Supplementary Figure S4 shows an example of these two encoding methods.

### 3.3. CrnnCrispr Model

**Figure 5:**
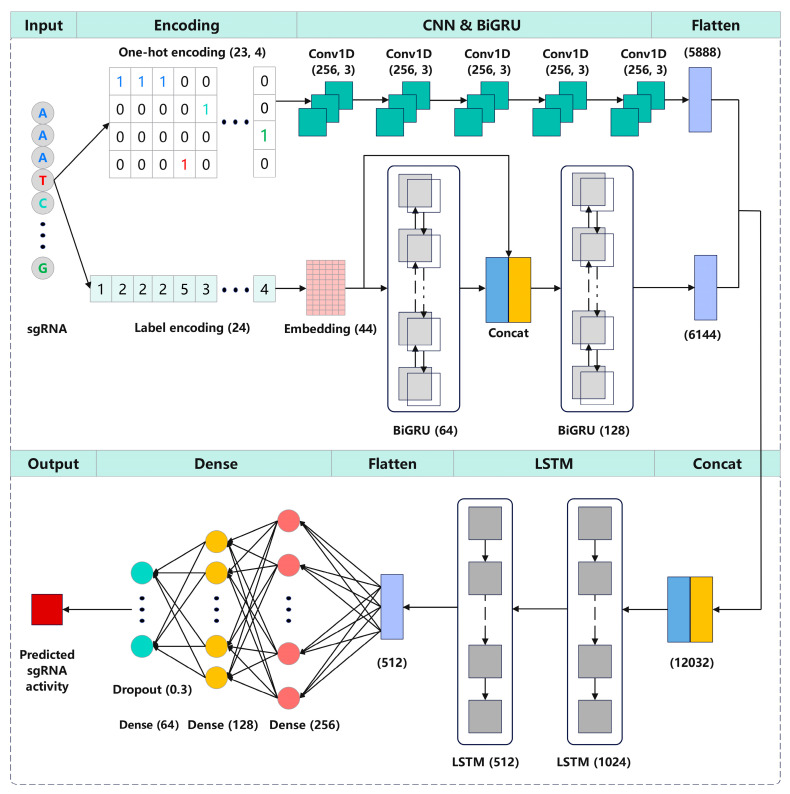
Illustration of the CrnnCrispr architecture. The sgRNA was first encoded by one-hot encoding and label encoding and was subsequently used as input of the CNN branch and BiGRU branch, respectively. The outputs of these two branches were concatenated and fed into two LSTM layers for dimensionality reduction. The outputs were flattened and input into three fully connected layers to generate the final representation. The outputs of the final fully connected layer were fed into a linear regression transformation to make a prediction of sgRNA on-target activity.

We propose CrnnCrispr, a hybrid neural network architecture, which combines a CNN and an RNN for sgRNA on-target activity prediction (Figure 5). The sgRNA was encoded into a 23 × 4 binary matrix through one-hot encoding, which was subsequently used as an input of the CNN branch. For the BiGRU branch, the sgRNA was encoded as a numeric sequence of length 24, which was then mapped to a dense high-dimensional space using an embedding layer and served as the input of BiGRU. These two branches can separately extract the high-dimensional features from each encoding, and the concatenation of the features at higher dimensions results in an effective feature interaction effect, thus helping our model to extract abundant features. The CNN branch consisted of five continuous one-dimensional convolutional layers, where each layer utilized a convolutional kernel with a size of 256 and a stride of 3 to extract local features between adjacent elements in the sgRNA sequence. Rectified linear units (ReLU) [38] were used as the activation function. The BiGRU branch consisted of two BiGRU layers with dimensions of 64 and 128, respectively. The outputs of the first BiGRU layer were concatenated and integrated with the output of the embedding layer before being fed into the next BiGRU layer. This process can enhance the correlation between the low- and high-dimensional features of the sequence while also incorporating both forward and backward information of the sgRNA sequence. The high-dimensional feature representations extracted from the two branches were concatenated and input into two consecutive LSTM layers to learn the directional and spatial relationships between motifs in sequences. After flattening, the output of the LSTM layer was fed into three consecutive fully connected layers, consisting of 256, 128 and 64 neurons, respectively. Additionally, dropout was used to regularize the model and prevent overfitting with a dropout rate of 0.3. The final output layer contained a single neuron for outputting the regression score, which is highly correlated with sgRNA activity.

### 3.4. Model Training

We implemented our CrnnCrispr model using Keras (2.4.3) and TensorFlow-GPU (2.5.0) as the backend, running on an Intel Xeon 4210R CPU (Intel, Senta Clara, CA, USA) with 2.4 GHz and 32 GB RAM and with an NVIDIA 12 GB RTX 3080TI GPU (MSI, New Taipei, China). We trained CrnnCrispr using Adamax [39] with the mean absolute error (MAE) [40] as the loss function. The formula of the *MAE* loss function is as follows:

(1) $$MAE=\overset{K}{\underset{n=1}{\sum }}\frac{\left|y_{true}-y_{prep}\right|}{k}$$

where *K* represents the number of test samples, and $y_{true}$ and $y_{prep}$ represent the sgRNA efficiency and predicted scores, respectively. Several methods have applied the mean squared error (MSE) [41] as the loss function for model training. Note that the MSE calculates the sum of squared distances between predicted values and true values, giving larger weights to outliers. Thus, it may lead to the problem of gradient explosion when there are significant differences between predicted and target values. In contrast, the MAE calculates the sum of the absolute differences between true values and predicted values, which is sensitive and stable for outliers. Considering that we selected three scales of datasets, we chose the *MAE* as the loss function.

The network parameters in CrnnCrispr, such as the number of neurons in each CNN layer, the size of the BiGRU layer and the learning rate, were determined based on our experience. Specifically, the choice of the value for the filters and kernel size was motivated by the success of CRISPRont. Additionally, we employed the value of the input dimension of the embedding layer as previously introduced in the work of [19]. We note that methods including TransCrispr, DeepSpCas9 and DeepCas9 applied a learning rate of 0.0001 to update the parameters. Inspired by these previous works, we used a fixed learning rate of 0.0001 and applied a grid search to choose the appropriate parameters of the training epoch and batch size. The following training hyperparameters were adjusted through the grid search: dropout rate over the choice (0.2, 0.3, 0.4 and 0.5), batch size over the choice (200, 300, 400 and 500) and number of epochs over the choice (100, 200, 300 and 400). The optimal hyperparameters were as follows: dropout rate of 0.3, batch size of 500 and epoch of 200. In addition, we employed the transfer learning strategy to improve the performance of our model on small-scale datasets. We used the benchmark dataset for model pretraining. The dataset was randomly divided into a training set and a testing set with the proportion of 8:2. We performed 5-fold cross-validation to select the optimal pre-trained model.

### 3.5. Performance Measurements

We used two regression question evaluation indicators, including Spearman’s correlation coefficient (*SCC*) [42] and Pearson’s correlation coefficient (PCC) [43], between the predicted sgRNA on-target activity scores and the ones in the public datasets to evaluate the performance of CrnnCrispr. *SCC* is defined as follows:

(2) $$SCC=1-\frac{6\sum_{i=1}^{n}{\left(X_{i}-Y_{i}\right)}^{2}}{n\left(n^{2}-1\right)}$$

where $X_{i}$ and $Y_{i}$ are the sorted $i^{th}$ elements in the sets of X and Y, and n represents the number of samples. *SCC* assesses the monotonic relationships between two variables. The sign of *SCC* represents the direction of association between  X and Y. If Y tends to increase when X elevates, *SCC* is negative. If there is no tendency for Y to either increase or decrease when X increases, *SCC* is zero. Thus, the value of *SCC* ranges from −1 to 1, with 1 and −1 indicating the strongest positive and negative correlation, respectively. The formula of *PCC* is as follows:

(3) $$PCC=\frac{\sum_{i=1}^{n}\left(X_{i}-\bar{X}\right)\left(Y_{i}-\bar{Y}\right)}{\sqrt{\sum_{i=1}^{n}{\left(X_{i}-\bar{X}\right)}^{2}}\sqrt{\sum_{i=1}^{n}{\left(Y_{i}-\bar{Y}\right)}^{2}}}$$

where $\bar{X}$ is the sample mean for X, and $\bar{Y}$ is the sample mean for Y. *PCC* measures the degree of linear correlation between two variables. A stronger correlation is denoted by an increased absolute value. *SCC* and *PCC* were calculated using the Scipy library (https://scipy.org, accessed on 12 February 2024).

## 4. Conclusions

In this study, we proposed a hybrid neural network model architecture called CrnnCrispr for CRISPR/Cas9 sgRNA on-target activity prediction. Experiments on nine public datasets with three scales (e.g., large-scale, medium-scale and small-scale) showed that CrnnCrispr achieved superior performance compared to four advanced deep learning models (TransCrispr, CRISPRont, DeepSpCas9 and DeepCas9). Specifically, CrnnCrispr outperformed the other methods on the large-scale and medium-scale datasets in terms of accuracy and generalizability. We also incorporated a fine-tuning strategy to boost the predictive ability of CrnnCrsipr in dealing with small-scale datasets. In addition to the prediction accuracy, we used DeepSHAP to estimate the feature importance of sequence inputs, thus enhancing the interpretability of our model.

## Figures and Tables

**Table 1:** Summary of CrnnCrispr model variants.

Model	Architecture
CrnnCrispr-onehot	Only uses one-hot encoding to encode the sgRNA sequence and inputs the encoded matrix into both the CNN and BiGRU branches
CrnnCrispr-label	Only uses label encoding to encode the sgRNA sequence and inputs the encoded matrix into both the CNN and BiGRU branches
CrnnCrispr-w/oConv	Eliminates the CNN branch in the model
CrnnCrispr-w/oBiGRU	Eliminates the BiGRU branch in the model

**Table 2:** Performance comparison of CrnnCrispr with four model variants on WT dataset under 5-fold cross-validation.

Model	SCC	PCC
CrnnCrispr	0.867 ± 0.007	0.892 ± 0.007
CrnnCrispr-onehot	0.863 ± 0.005	0.885 ± 0.006
CrnnCrispr-label	0.863 ± 0.004	0.887 ± 0.004
CrnnCrispr-w/oConv	0.836 ± 0.005	0.865 ± 0.007
CrnnCrispr-w/oBiGRU	0.861 ± 0.006	0.884 ± 0.007

Note: CrnnCrispr-w/oConv is a variant that does not use the convolutional module. CrnnCrispr-w/oBiGRU is a variant does not use the BiGRU module. SCC and PCC values between sgRNA efficiency scores and predicted scores are shown. Performance is shown as mean ± standard deviation (SD).

**Table 3:** Performance comparison of CrnnCrispr and four existing deep-learning-based models on three cell line datasets using fine-tuning under 5-fold cross-validation.

Method	HCT116	HELA	HL60	Average
(a) SCC				
CrnnCrispr	0.768 ± 0.055	0.709 ± 0.055	0.545 ± 0.049	0.674 ± 0.053
TransCrispr	0.629 ± 0.019	0.625 ± 0.020	0.535 ± 0.050	0.596 ± 0.030
CRISPRont	0.765 ± 0.067	0.706 ± 0.052	0.527 ± 0.030	0.666 ± 0.050
DeepSpCas9	0.646 ± 0.031	0.615 ± 0.041	0.471 ± 0.035	0.577 ± 0.036
DeepCas9	0.698 ± 0.027	0.667 ± 0.026	0.518 ± 0.043	0.628 ± 0.032
(b) PCC				
CrnnCrispr	0.732 ± 0.053	0.633 ± 0.049	0.541 ± 0.047	0.635 ± 0.050
TransCrispr	0.633 ± 0.024	0.595 ± 0.019	0.561 ± 0.031	0.596 ± 0.025
CRISPRont	0.735 ± 0.053	0.649 ± 0.042	0.509 ± 0.023	0.631 ± 0.039
DeepSpCas9	0.548 ± 0.026	0.506 ± 0.043	0.341 ± 0.053	0.465 ± 0.041
DeepCas9	0.649 ± 0.035	0.598 ± 0.033	0.499 ± 0.057	0.582 ± 0.042

Note: Performance is shown as mean ± SD.

**Table 4:** Dataset used for this study.

Dataset	Training	Validation	Testing	Total	Scale Level	Ref.
Benchmark	115,528	28,882	36,102	180,512	Large	[25]
WT	42,536	4726	8341	55,603	Large	[27]
ESP	44,841	4982	8793	58,616	Large	[28]
HF	43,519	4835	8533	56,887	Large	[29]
xCas	28,869	3208	5661	37,738	Medium	[30]
SpCas9	23,397	2600	4588	30,585	Medium	[31]
Sniper	28,912	3213	5669	37,794	Medium	[32]
HCT116	3243	360	636	4239	Small	[33]
HELA	6197	689	1215	8101	Small	[33]
HL60	1588	177	311	2076	Small	[34]

## Data Availability

Data is contained within the article.

## Supplementary Materials

The following supporting information can be downloaded at: https://www.mdpi.com/article/10.3390/ijms25084429/s1.
