# Supplementary material for: CrnnCrispr: An Interpretable Deep Learning Method for CRISPR/Cas9 sgRNA On-Target Activity Prediction
Source: Int J Mol Sci. 2024 Apr 17;25(8):4429. doi: 10.3390/ijms25084429 (PMC11050447; doi:10.3390/ijms25084429)
Supplement: Supplementary file 1 [file ijms-25-04429-s001.zip › ijms-2936602-supplementary.pdf]

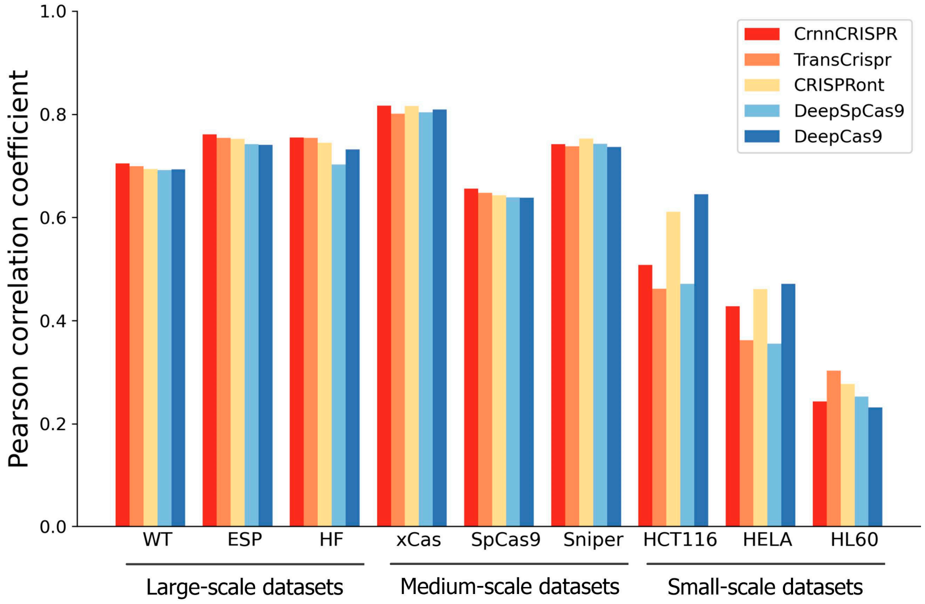

Figure S1. Performance comparison in terms of PCC of CrnnCrispr and four existing deep learning-based methods on nine datasets with various scales under a leave-one-cell-out procedure.

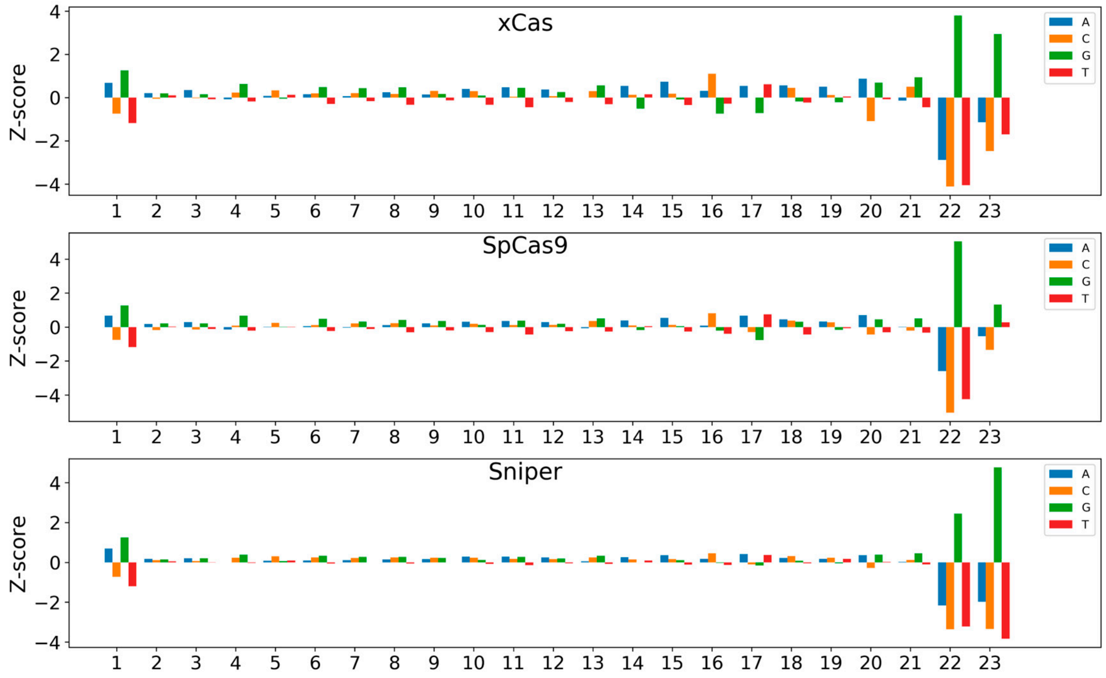

Figure S2. Impact of nucleotide composition of sgRNA activity on three medium-scale datasets. Bars show the Z-scores of nucleotide frequency for each position. The numbers below represent the positions of the sequence.

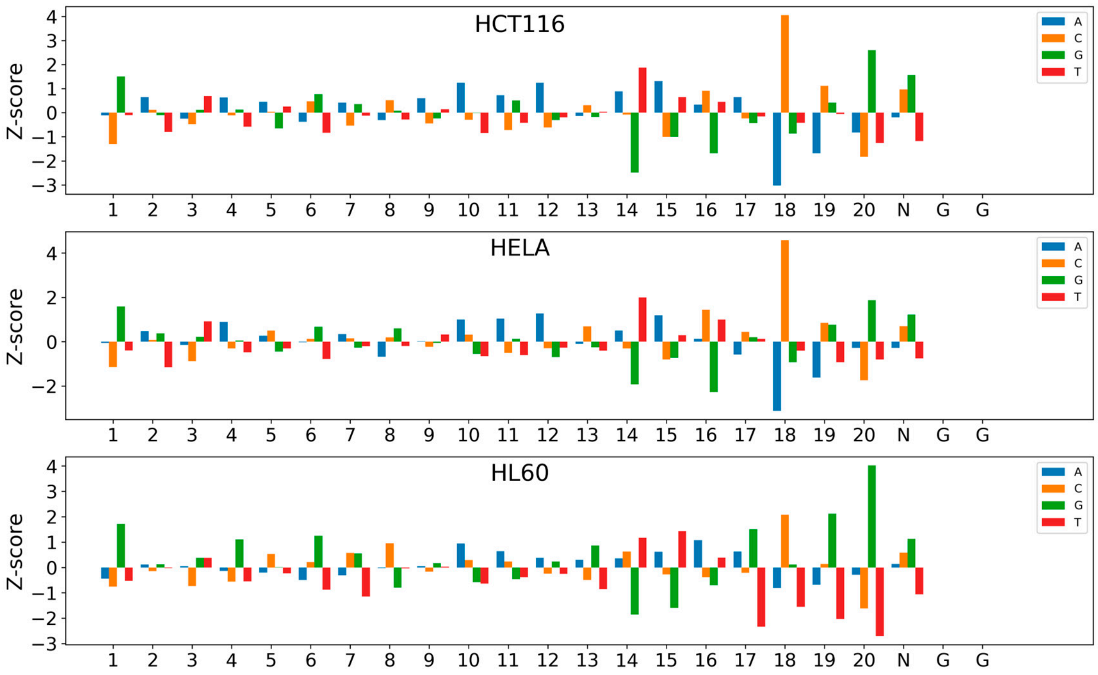

Figure S3. Impact of nucleotide composition of sgRNA activity on three small-scale datasets. Bars show the Z-scores of nucleotide frequency for each position. The numbers below represent the positions of the sequence.

	A	A	A	A	T	C	A	A	G	A	C	A	T	G	G	G	C	A	C	T	G	G	G
1	2	2	2	2	5	3	2	2	4	2	3	2	5	4	4	4	3	2	3	5	4	4	4

Label Encoding

A	1	1	1	1	0	0	1	1	0	1	0	1	0	0	0	0	0	1	0	0	0	0	0
C	0	0	0	0	0	1	0	0	0	0	0	0	0	0	0	0	1	0	1	0	0	0	0
G	0	0	0	0	0	0	0	0	1	0	1	0	0	1	1	1	0	0	0	0	1	1	1
T	0	0	0	0	1	0	0	0	0	0	0	0	1	0	0	0	0	0	0	1	0	0	0
	**1**	**2**	**3**	**4**	**5**	**6**	**7**	**8**	**9**	**10**	**11**	**12**	**13**	**14**	**15**	**16**	**17**	**18**	**19**	**20**	**21**	**22**	**23**

One-hot Encoding

Figure S4. An example of the one-hot encoding and label encoding representation schema for sgRNA.
